# Deconstructing the driving factors of land development intensity from multi-scale in differentiated functional zones

**DOI:** 10.1038/s41598-022-20935-9

**Published:** 2022-10-10

**Authors:** Yue Yin, Kan Zhou, Yufan Chen

**Affiliations:** 1 Key Laboratory of Regional Sustainable Development Modeling, Institute of Geographic Sciences and Natural Resources Research, Chinese Academy of Sciences, Beijing, 100101, China; 2 University of Chinese Academy of Sciences, Beijing, 100049, China

**Keywords:** Environmental sciences, Environmental social sciences

## Abstract

Land development intensity (LDI) is an important indicator of how much human exploitation of land resources. Against the background of rapid urbanization and industrialization, in order to curb the over-occupation of agricultural and ecological space by urbanized space, China has proposed the Major Functional Zoning Planning (MFZP) as a new spatial governance model, which attempts to implement differentiated LDI control according to the functional positioning of different regions. To observe the spatio-temporal evolution and drivers of LDI in the first five years since the implementation of the MFZP, we chose the Yangtze River Delta, the most urbanized region in China, as a case area. The multi-scale distribution pattern of LDI was analyzed by combining macro and micro scales, and a new spatial measurement method integrating global and local regression models was developed to quantitatively deconstruct the natural-human drivers of LDI in different functional zones. Results demonstrate that LDI decreases in a gradient of the urbanized zone (UZ) > main agricultural production zone (MAPZ) > key ecological function zone (KEFZ). In the UZ, LDI is influenced by natural-human multi factors, e.g., terrain conditions (SLP), proportion of cultivated land area (PCL), proportion of ecologically important area (EID), population urbanization rate (PUR), GDP per capita (PGDP), fixed asset investment size per land (FAI) and fiscal expenditure as a percentage of GDP (FD). The LDI in the MAPZ is mainly affected by the facilitating role of PCL, EID, FAI, and the prominent role of FD, while that in the KEFZ is mainly inhibited by SLP, EID, and driven by the PGDP. The diverse drivers of LDI in different functional zones remind us to implement differentiated spatial control according to functional positioning and to adopt refined policy tools of zoning and classification to ensure that land resources within each type of functional zone can be used sustainably.

## Introduction

Since 2010, China has stepped into the transition of industrialization and urbanization development, with the urbanization rate growing rapidly from 49.7% in 2010 to 51.3% in 2011, officially breaking the 50% mark. More importantly, in the National 11th Five-Year Plan in 2010, China formally proposed the Major Functional Zoning Planning (MFZP). MFZP is the first hierarchical control based on geographical functions at the county scale in the history of China, forming four typical zones, i.e., the optimized development zones (ODZ) with strict control requirements for construction land increment; prioritized development zones (PDZ) with the relatively appropriate expansion of the construction land; main agricultural production zone(MAPZ) focusing on agricultural development and construction; key ecological function zone (KEFZ) where the change of ecological land use is strictly prohibited and the construction land is basically zero growth. ODZ and PDZ can be further combined as an urbanized zone (UZ). The MFZP greatly affects the existing land development patterns and land use methods. Land development intensity (LDI) refers to the proportion of construction land within the administrative area and is often used in territorial spatial planning. As Western countries entered a phase of rapid industrialization in the latter half of the twentieth century, some researchers conducted preliminary studies on land management and territorial development in response to the city expansion and rapid urbanization^1,2^. Research on LDI in China started in the 1980s after the government put forward the instruction on national land improvement^3–6^. Later, in the 12th Five-Year Plan, the LDI has been proposed officially^7^. Coupled with the environmental pollution and the imbalance in development that has emerged in land construction, the study of the LDI has gradually become a hot topic since 2010.

Existing research has shown that there is a shift in the concept of the LDI. The general concept of LDI is the area ratio of the construction land to the total administrative region^8^. With the over-development of arable land resources affecting food security and waste caused by inefficient urban development, more researchers were aware of the harmonious relationship between land development and other socio-economic and ecological factors, making the concept of LDI change from a broad to a narrow sense, highlighting the combination of human and natural elements. The narrow sense of LDI more focuses on the ratio of regional construction land scale to the area of land suitable for construction in natural conditions^9^. Later, many researchers measured the spatio-temporal variation of the LDI at the national, provincial, city cluster, and municipal scales, and spatially portrayed it through different scales^8,10–13^.

It is equally important to find the drivers that may affect the LDI and its spatial distribution. Against the background of urbanization and industrialization, the main factors affecting the LDI were still biased towards economic development, population size, and industrial structure^13–15^. Moreover, the direct or indirect effects of financial investments and policies on LDI have also been initially revealed in the study^16,17^. Researchers mostly used global regression (e.g., ordinary least squares, OLS) or local regression (e.g., geographically weighted regression, GWR) to estimate the natural and social factors affecting LDI, as well as their spatial effects^14,18^. Currently, part of the research on LDI was combined with resource and environmental carrying capacity and then widely used in the land spatial planning application^19^. After the MFZP was put forward, China's regional development and spatial structure adjustment strategies became more differentiated^20^. Based on this policy, the research has combined the LDI with ecological environmental protection, spatial order maintenance, etc., and proposed a higher quality, more efficient and higher output per unit development process^21–23^. The LDI is playing an increasingly important role in China's territorial development system.

Research on the spatio-temporal evolution of LDI and its drivers has become a hot spot in academia. However, there is still something that needs to be deepened. (1) The LDI, as an important indicator for administrative districts to control the way of spatial development and utilization within their jurisdictions and a key target parameter for spatial planning at all scales, has distinctive multi-spatial scale characteristics in space in China. Based on the spatio-temporal distribution of LDI at provincial and municipal levels, it is urgent to explore the distribution and drivers of LDI at the county and even functional zone scales in a more detailed way and to establish a new research framework for LDI that systematically deconstructs the global and local spatial characteristics by combining macro and micro scales. (2) Related research on land use and land cover change focuses on natural-type drivers that impact the LDI, while land economists focus more on human-type drivers. However, mining the driving factors of LDI from a single dimension is often not comprehensive. It is necessary to strengthen the composite analysis of the natural and human drivers, integrate multiple analytical dimensions such as natural, socio-economic, and policy, and quantitatively reveal the strength of the drivers. (3) The proposal of major policies should have an impact on urban development and construction. In the context of implementing differentiated land management policies according to the functional positioning of the major functional zoning, it is more important to pay attention to the spatio-temporal response of LDI after the new model of county-scale zoning management is proposed, examine the effectiveness of the model on LDI control and adjust policy tools promptly. Thus, we choose the Yangtze River Delta (YRD) as the case area, analyze the spatio-temporal evolution of the LDI from the multi-scale of "provincial–municipal–county–functional zone" in the first-five-year period for the MFZP implementation (i.e., 2011–2016), and then use global and local regression models to calculate the natural-human drivers of the LDI in different functional zones and assess the policy effectiveness of the MFZP.

## Methods

### Study area and data sources

**Figure 1:**
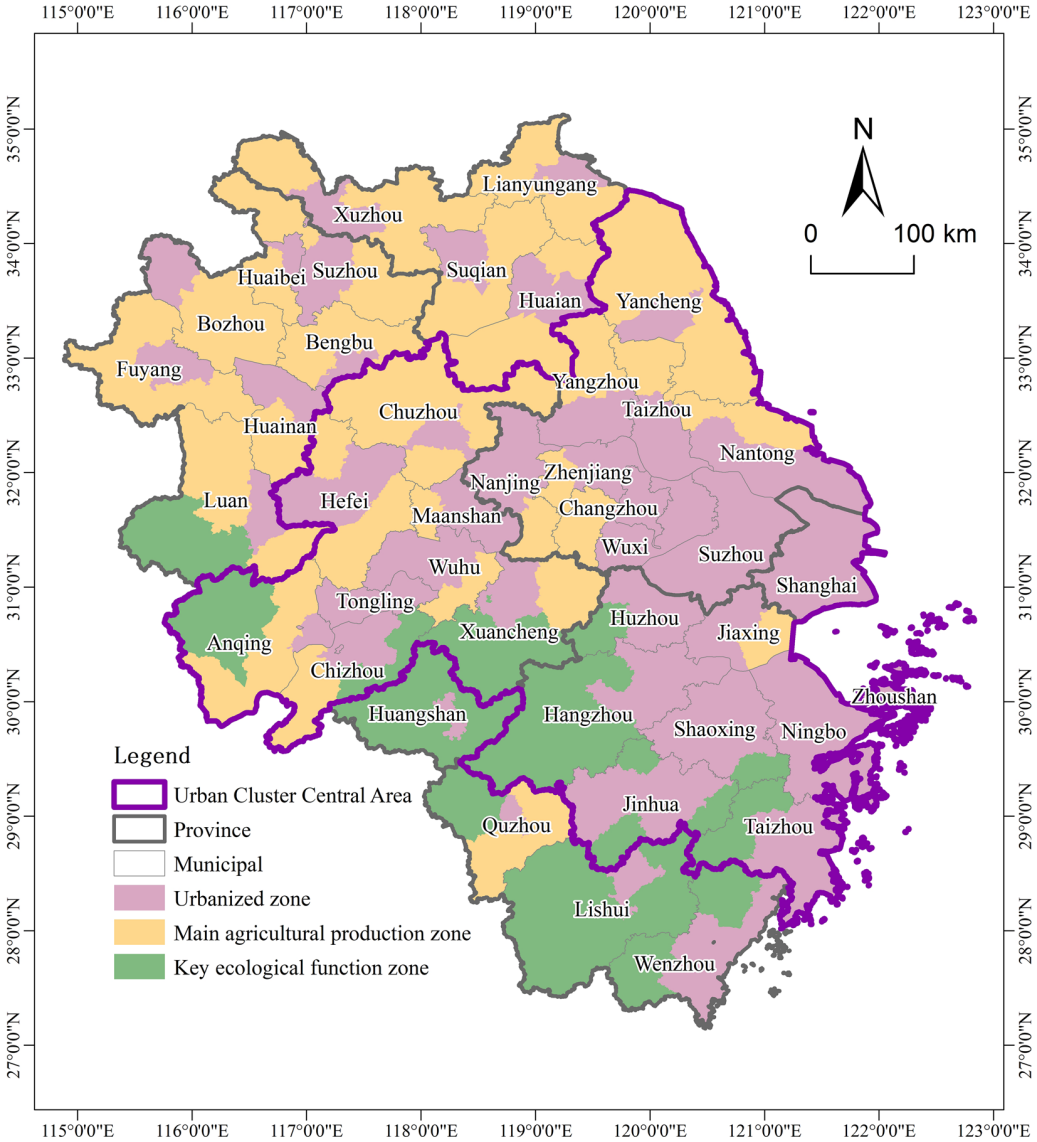
Scope of case area. Software: Arcgis 10.7. URL: https://www.esri.com/zh-cn/home.

The YRD consists of three provinces and a city (i.e., Jiangsu, Zhejiang, Anhui, and Shanghai), involving 41 cities and 305 counties. Further, the YRD has built a hierarchical scale structure of towns and cities of super cities, mega-cities, large, medium and small cities, and then presents a spatial form of ‘one core, five sub-cores’ as urban agglomerations. Since the MFZP was proposed, the YRD has entered a time of rapid land urbanization with an average annual growth rate of 1.91% during 2011–2016. Based on the major functional positioning of counties in the YRD, three types of functional zones have been formed, i.e., UZ, MAPZ and KEFZ (Fig. 1). UZ has 186 counties which are mainly located in the eastern coastal areas and the center of the city. MAPZ has 78 counties which are located in northern Jiangsu and Anhui. KEFZ has 41 counties which are located in low hill counties of southern Anhui and western Zhejiang.

We use county-level administrative districts as the basic statistical unit to construct a database on LDI and socio-economic foundations from 2011 to 2016. The main data and sources for this are: Basic geographic information data, such as administrative divisions and topography at all levels, was obtained from the China National Basic Geographic Information System website (http://www.ngcc.cn/ngcc/). Land use data was obtained from the natural resources department. Socio-economic data was mainly obtained from *the China County Statistical Yearbook *(https://data.cnki.net/trade/Yearbook/Single/N2013020080?zcode=Z022),* the Shanghai Statistical Yearbook *(https://data.cnki.net/area/Yearbook/Single/N2012080020?dcode=D09),* the Jiangsu Statistical Yearbook *(https://data.cnki.net/area/Yearbook/Single/N2012090083?dcode=D10),* the Zhejiang Statistical Yearbook *(https://data.cnki.net/area/Yearbook/Single/N2012100015?dcode=D11)*, the Anhui Statistical Yearbook *(https://data.cnki.net/area/Yearbook/Single/N2012100009?dcode=D12)* and the County National Economic and Social Development Bulletins and Statistical Yearbook.* It should be noted that Pingjiang, Canglang and Jinchang underwent administrative division adjustments in 2011–2016, which affected the LDI of Hefei, Wuhu and Maanshan, and we have merged and adjusted the data accordingly.

### Research methodology

#### Global regression model

A global regression model uses a global linear or non-linear regression relationship between the target variable and one or more variables to make predictions, and the results of the OLS do not take spatial weights into account. To investigate the degree of interdependence between LDI in each county and to incorporate spatial correlation and spatial heterogeneity into their driver analysis, we used the Spatial Lag Model (SLM) and the Spatial Error Model (SEM) to analyze the spatial autocorrelation regression^24,25^. The SLM, in which the spatial interaction is assessed using a spatial lag term to analyze the spatial spillover effect of LDI of counties on adjacent counties, is formulated as Eq. (1):

1 $$Y=\rho {{\varvec{W}}}_{{\varvec{y}}}+\beta {\varvec{X}}+\varepsilon$$

where *Y* is the explained variable, *X* is the matrix of exogenous explanatory variables, *ρ* is the spatial regression coefficient to reflect the degree of spatial dependence, *β* is the coefficient to be estimated, ${{\varvec{W}}}_{{\varvec{y}}}$ is the spatially lagged dependent variable and *ε* is the random error vector. While the SEM uses a spatial error term to measure the magnitude of the impact of error shocks to the explained variables on adjacent county cells, it’s a model formulation in Eq. (2):

2 $$Y=\beta {\varvec{X}}+\varepsilon , \varepsilon =\lambda {{\varvec{W}}}_{{\varvec{\varepsilon}}}+\mu$$

where *λ* is the spatial error coefficient to reflect the degree of spatial dependence present in the error term, ${{\varvec{W}}}_{\varepsilon }$ is the spatial weight matrix of the error term and *μ* is the white noise.

#### Local regression model

To analyze the local effects of the drivers for LDI in a spatial region, we adopted a Multiscale Geographically Weighted Regression (MGWR) model to measure them. The MGWR allows for changes in the relationship between variables and explanatory variables at spatial and different scales in response^26,27^, thus better exploring the scale effects and spatial differentiation of the drivers of LDI. The formula is as follows:

3 $${y}_{i}=\sum_{j=1}^{k}{\beta }_{bij}\left({u}_{i},{v}_{i}\right){x}_{ij}+{\varepsilon }_{i}$$

where *β*_*bij*_ denotes the regression coefficient of the local variable, *b*_*ij*_ denotes the bandwidth used for the regression coefficient of variable *j*, and (*u*_*i*_,*v*_*i*_) represents the spatial coordinates of sampling point *i*, *x*_*ij*_ is the observation of variable *j* at sampling point *i*, while *ε*_*i*_ is the random disturbance term. Each regression coefficient β of MGWR is obtained based on local regression and the bandwidth possesses heterogeneity. The kernel function and bandwidth criteria for the MGWR in this paper use the quadratic kernel function and the AICc criterion.

#### Variable settings and descriptions

We selected the LDI as the explained variable to study 305 counties in the YRD from 2011 to 2016. LDI is the ratio of the total amount of built-up land to the area of the administrative district, reflecting land development in the spatial control system of the country and an important parameter in the planning strategy and monitoring of the major functional zones.

Like the explanatory variables listed in Table 1, the terrain condition (SLP) represents the mean value of slope and characterizes the natural background conditions^18^. The proportion of cultivated land area (PCL) represents the proportion of cultivated land, indicating the pressure on cultivated land protection and the ability of land resource conditions to support the construction activities in the county^28^. The proportion of ecologically important area (EID) is an evaluation of the importance of ecosystems based on natural units and is the proportion of the important areas to the county territory^18,29^. Population urbanization rate (PUR) represents the level of urban construction in the county and is expected to reveal its positive contribution to LDI^17,30^. GDP per capita (PGDP) characterizes the level of economic development of the county, which is usually positively driven by the level of economic development on LDI^11,12^. Fixed asset investment size per land (FAI) characterizes the intensity of investment in land in the county, and capital investment in land development drives economic development while creating a demand for building space, but is also influenced by the structure of the investment sector^31–33^. Fiscal expenditure as a share of GDP (FD) indicates the strength of the county's fiscal investment. With the intensive development and use of land space, the local government can regulate the fiscal control, so that the original positive effect on the expansion of land from a large area to the negative effect of reasonable restrictions on excessive development^15^.

**Table 1:** Descriptive statistics of main variables.

Variable type		Variables	Mean	Std	Predicted role
Explained variable		Land development intensity (LDI)	25.1	21.68	–
			26.25	21.68	
Explanatory variables	Natural factors	Terrain conditions (SLP)	5.7	6.89	Negative
		The proportion of cultivated land area (PCL)	31.89	19.32	Positive or negative
			31.70	19.18	
		The proportion of ecologically important area (EID)	1.57	0.49	Negative
	Human factors	Population urbanization rate (PUR)	50.91	35.99	Positive
			67.05	21.85	
		GDP per capita (PGDP)	55,654.09	48,624.38	Positive
			76,902.1	69,024.62	
		Fixed asset investment size per land (FAI)	19,773.15	11,661.67	Positive or negative
			39,002.68	24,982.32	
		Fiscal expenditure as a share of GDP (FD)	12.67	6.84	Positive or negative
			17.25	15.44	

## Results

### Spatio-temporal characteristics of LDI in the YRD region

#### Spatio-temporal characteristics at the provincial level

**Figure 2:**
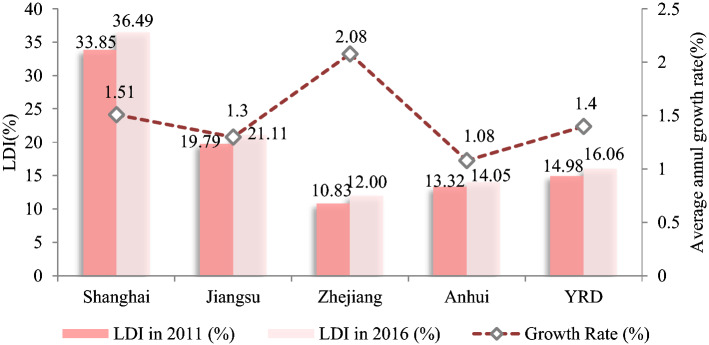
LDI in the YRD from 2011 to 2016. Software: Excel 2010. URL: https://www.microsoft.com/zh-cn/.

From 2011 to 2016, the LDI in the YRD showed a steady increase, rising from 14.98% to 16.06% at a rate of 1.40%. It basically matched the land urbanization rate of 1.91% and the population urbanization rate of 1.72% across the region in the same period, indicating the coordinated development of land construction in the YRD region and the gathering of the population into cities and towns. LDI had a significant provincial divergence, with a development gradient of Shanghai > Jiangsu Province > Anhui Province > Zhejiang Province (Fig. 2). Jiangsu, Anhui, and Shanghai have remained relatively flat over the five years, while Zhejiang was in a period of significant acceleration. Zhejiang has a large proportion of gardens and forests, accounting for 59.17% of the total land. Due to the natural state, the LDI in Zhejiang was relatively low, but it was still in the process of rapid land development. Anhui is mostly hilly and mountainous with undulating terrain, which causes its LDI to be at a relatively low level and grow slowly. In contrast, Shanghai and Jiangsu have a large base of land construction and both have been in a slow-growth phase over the five years.

#### Spatio-temporal characteristics at the municipal level

**Figure 3:**
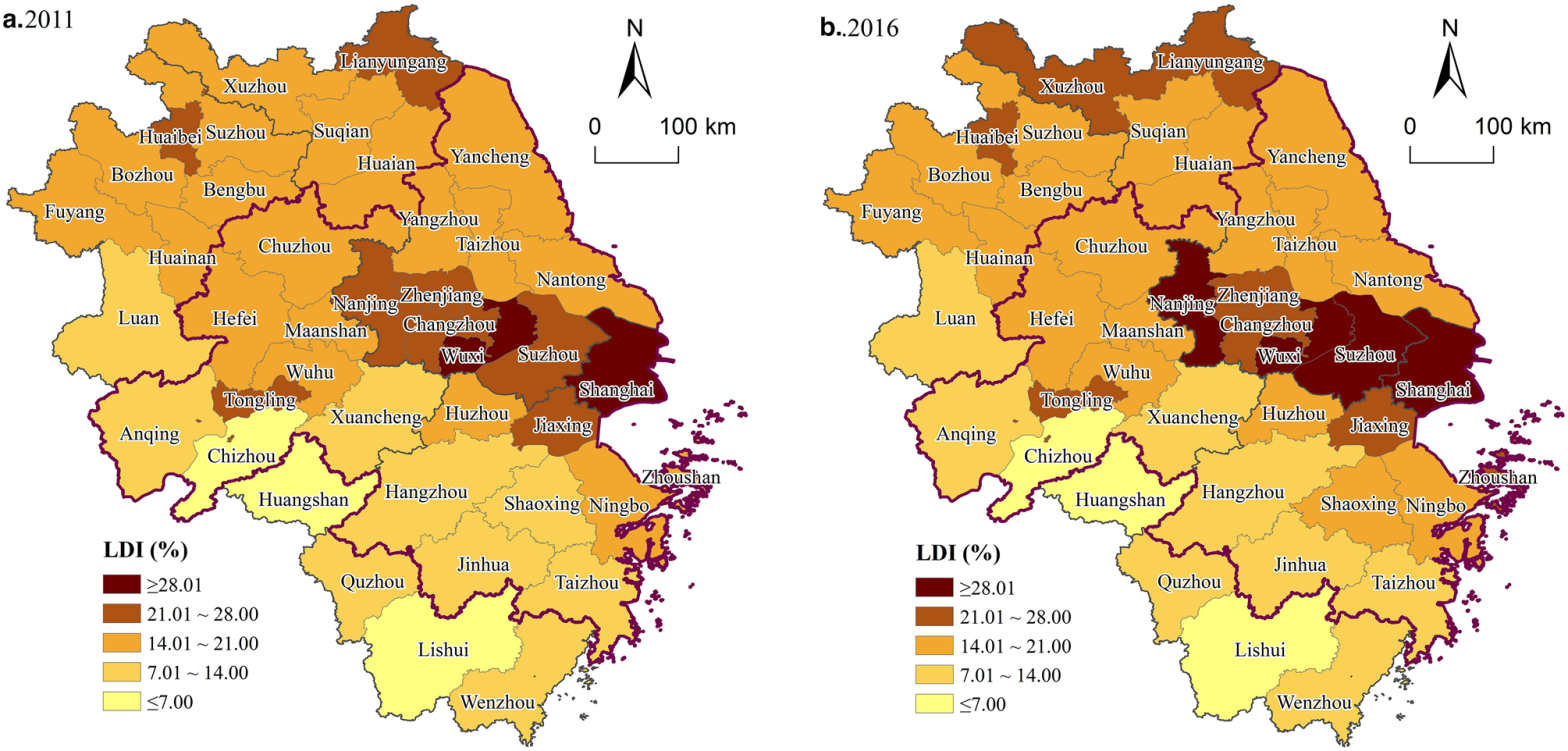
The spatial distribution of the LDI at the municipal level in YRD. (**a**) 2011; (**b**) 2016. Software: Arcgis 10.7. URL: https://www.esri.com/zh-cn/home.

**Figure 4:**
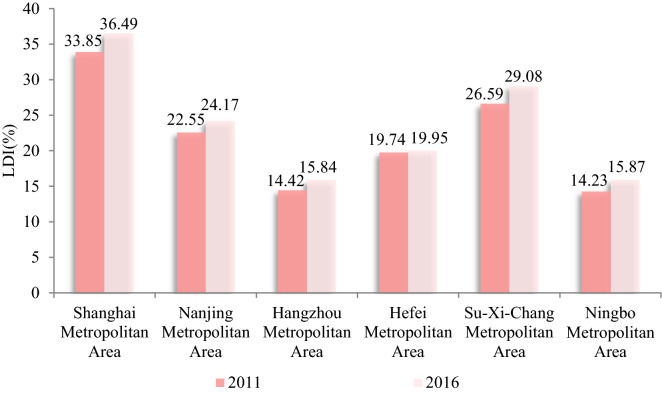
Changes in LDI in the metropolitan area of the YRD in 2011 and 2016. Software: Excel 2010. URL: https://www.microsoft.com/zh-cn/.

The average LDI in the municipalities increased from 16.98% in 2011 to 18.16% in 2016, with an average annual increase of 1.36%. As shown in Fig. 3, the LDI in the central area of the city cluster raised from 18.38% to 19.81%, with an average annual increase of 1.51%, which was remarkably higher than the average LDI across the region. Of these, the LDI of Shanghai metropolitan area, Su-Xi-Chang metropolitan area, Nanjing metropolitan area, Hefei metropolitan area, Ningbo metropolitan area, and Hangzhou metropolitan area were 36.49%, 29.08%, 24.17%, 19.95%, 15.87% and 15.84% in 2016 (Fig. 4). It shows that the main urban agglomeration in the YRD, especially the inner circle (i.e., one core and five sub-cores), is an important driving force for land construction activities, and the development pattern and the land construction agglomeration effect within each metropolitan area is driven by the core cities (e.g., Nanjing, Jiaxing, Hefei, Wuxi, and Zhoushan).

#### Spatio-temporal characteristics at the county level

**Figure 5:**
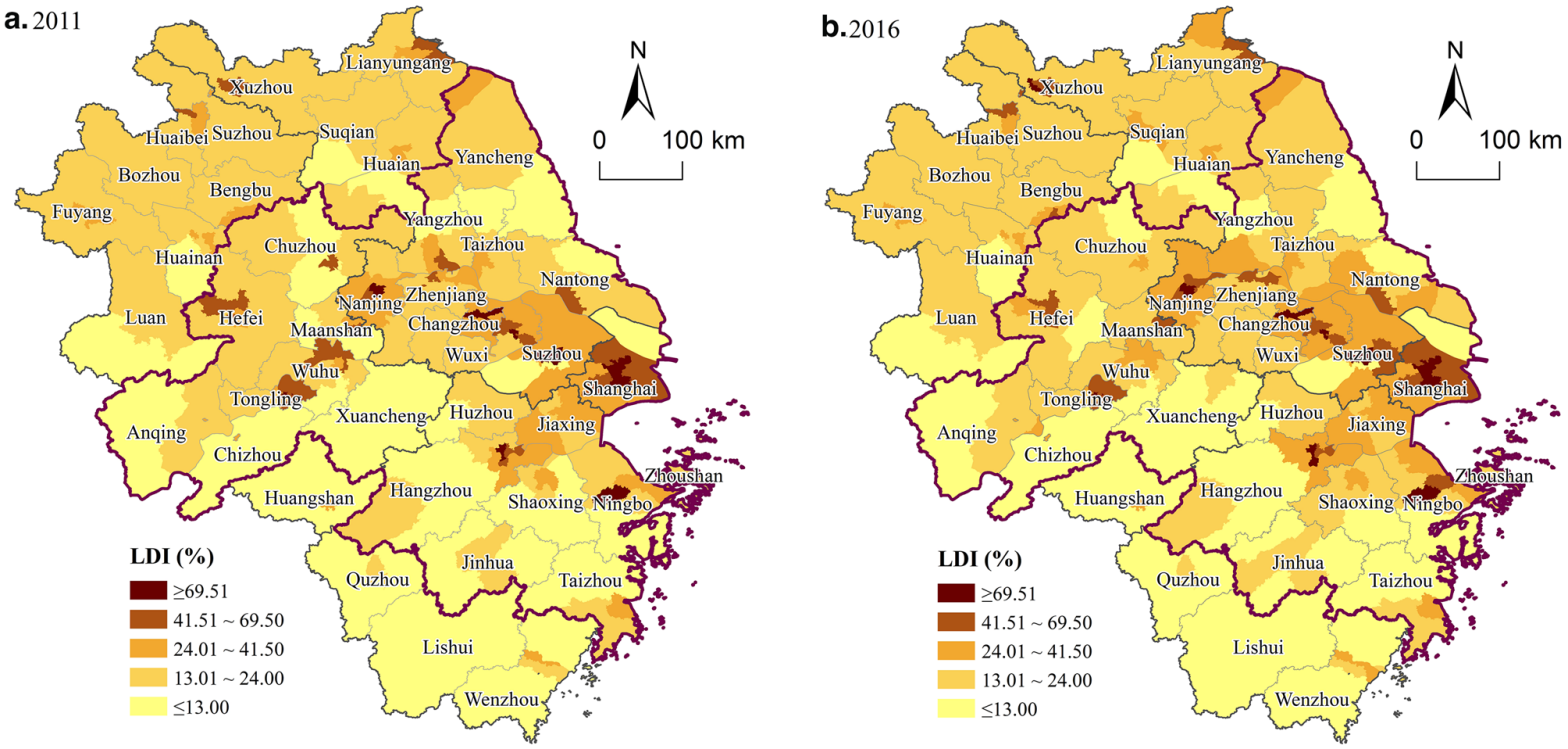
The spatial distribution of the LDI at the county level in YRD. (**a**) 2011; (**b**) 2016. Software: Arcgis 10.7. URL: https://www.esri.com/zh-cn/home.

The average LDI in the counties of the YRD increased from 25.10% in 2011 to 26.25% in 2016, with an average increase of 5.83% over the five years. The LDI at the county level in the YRD was generally on the rise and shows significant spatial variation. It can be seen from Fig. 5 that the high-value counties of LDI were mainly concentrated in central areas of the central city and driven by the radiation of the province and city cluster, the integrated development of construction land in adjacent counties is remarkable, especially in Xuzhou and Suzhou. In contrast, the LDI of the outer suburban counties was relatively low, especially in the KEFZ, showing a core-periphery structure and reflecting the role of ecological function positioning in guiding the LDI in the YRD.

#### Spatio-temporal characteristics at the functional zone level

The LDI decreased region by region according to a gradient of UZ > MAPZ > KEFZ (Table 2). The UZ is mainly located in Shanghai-Nanjing-Hangzhou-Ningbo along the river counties, where the overall LDI is high and has increased rapidly from 33.39% in 2011 to 34.91% in 2016. The economic effects of the Shanghai center radiate to the Su-Xi-Chang and Ningbo metropolitan areas, and the large concentration of population, capital, technology, and strong intrinsic links keep the sub-region in a state of high-intensity development. However, the counties within the UZ also show a clear geographical divergence. Relying on the advantages of water transport and economic activity, counties along the river, such as Tongling, Wuhu, Nanjing, Shanghai, Ningbo, and Jiaxing, develop at a higher rate than inland areas such as northern Jiangsu, southern Zhejiang, and Anhui. The MAPZ is mainly located in northern Jiangsu and northern Anhui, where the LDI has developed most steadily, with an average growth rate of 0.78%. 65% of the counties have growth rates concentrated between 0.00 and 1.00%, which is related to the long-term stability of agricultural development under the demands of natural conditions. The KEFZ is located in the south of Anhui and northwest of Zhejiang. Constrained by natural conditions and environmental policies, the regional development intensity base is small, but the growth rate is rapid, with an average annual growth rate of up to 1.57%.

**Table 2:** LDI of each province (city) in different major functional zones.

Major functional zones	Shanghai		Jiangsu		Zhejiang		Anhui	
	2011	2016	2011	2016	2011	2016	2011	2016
Urbanized zone	66.39	67.04	37.82	39.66	26.23	27.69	27.87	28.46
Main agricultural production zone	–	–	16.40	17.14	14.63	15.90	15.02	15.47
Key ecological function zone	–	–	–	–	5.83	6.31	5.13	5.53

### Multi-scale deconstruction of driving factors

#### Analysis of global regression estimation

The OLS test (Table 3) showed significant spatial lag and spatial error effects with the great likelihood LM-Lag and LM-Error tests being significant. The Robust LM-Lag and Robust LM-Error tests showed that the Robust LM-Lag was only significant through 10% in 2011 and the Robust LM-Lag was significant in 2016, in summary, the SEM model fitted better than the SLM model during 2011–2016. Therefore, we chose the SEM to estimate the global spatial correlation quantitively.

**Table 3:** Model spatial correlation test results.

Test indicators	2011		2016	
	Statistical quantities	Probability	Statistical quantities	Probability
Moran’s I(error)	12.1207	0.0000	9.0822	0.0000
LM-lag	62.0376	0.0000	56.4350	0.0000
Robust LM-lag	2.7945	0.0946	12.2090	0.0005
LM-error	129.2170	0.0000	71.4882	0.0000
Robust LM-error	69.9739	0.0000	27.2622	0.0000

The results of the SEM based on the GeoDa platform showed that natural-human factors such as SLP, PCL, EID, PGDP, FAI, and FD all passed the 1% significance test, except for the PUR in 2016 (Table 4). The parameter estimation results showed that the PUR and PGDP had a stable and significant positive drive on LDI during 2011–2016, while SLP, PCL, EID, FAI, and FD had significant negative effects. The positive effect of PUR was the most evident, with the elasticity coefficients of lnPUR being 0.326 and 0.155 for 2011 and 2016. It reflects that the concentration of population in towns and cities is the dominant factor influencing land expansion and construction activities. PGDP conducted the LDI to a lower extent, with coefficients of 0.156 and 0.292. The coefficients of lnSLP and lnEID were both negative in 2011 and 2016, indicating that land development activities are constrained by natural topographic conditions and the importance of ecosystems. The coefficients of lnPCL on LDI were − 0.242 and − 0.197, which reflects that the smaller PCL, the greater the pressure on the county's cultivated land resources, and the higher the LDI. The coefficient of lnFAI decreased from − 0.151 to − 0.223, indicating a further dampening effect of fixed asset investment efforts on land development. The coefficient of the lnFD was all in the range from − 0.245 to − 0.255, reflecting a stable inhibitory effect of local fiscal regulation on LDI.

**Table 4:** Verification and parameter estimation results of SEM.

*Variables*	2011		2016	
	Coefficient	Probability	Coefficient	Probability
*lnSLP*	− 0.179***	0.000	− 0.178***	0.000
*lnPCL*	− 0.242***	0.000	− 0.197***	0.000
*lnEID*	− 1.093***	0.000	− 1.030***	0.000
*lnPUR*	0.326***	0.000	0.155**	0.017
*lnPGDP*	0.156***	0.001	0.292***	0.000
*lnFAI*	− 0.151***	0.000	− 0.223***	0.000
*lnFD*	− 0.248***	0.000	− 0.253***	0.000
Spatial error term (λ)	0.697***	0.000	0.643***	0.000
R^2^	*0.866*		*0.855*	
log	− *89.781*		− *89.040*	
AIC	*195.561*		*194.08*	
SC	*225.324*		*223.842*	

***p < 0.01, **p < 0.05, *p < 0.1, t-statistics in parentheses.

Italic values are the model goodness of fit, reflecting the basic information and model fit.

#### Analysis of local regression estimation

We used the goodness of fit to measure how well the regression values fit the observations. From Table 5, it is easy to find that the model-adjusted goodness of fit was > 0.89 for both 2011 and 2016, and it can be judged that the regression model performs better and the study has scientific validity. The PGDP and FAI were global variables in 2011 (i.e., bandwidth/number of samples > 90%), and the rest were local variables. While in 2016, only the PGDP and EID were global variables.

**Table 5:** The statistical description of the MGWR coefficient.

MGWR		2011				2016			
		Mean	Std	Min	Max	Mean	Std	Min	Max
Variables	*lnSLP*	− 0.124	0.072	− 0.224	− 0.007	− 0.210	0.273	− 0.661	0.175
	*lnPCL*	− 0.242	0.205	− 0.498	0.209	− 0.249	0.215	− 0.645	0.335
	*lnEID*	− 0.388	0.148	− 0.647	0.092	− 0.351	0.010	− 0.369	− 0.332
	*lnPUR*	0.478	0.246	0.119	0.900	0.129	0.057	0.047	0.227
	*lnPGDP*	0.201	0.010	0.185	0.216	0.377	0.005	0.369	0.386
	*lnFAI*	− 0.092	0.015	− 0.124	− 0.066	− 0.118	0.057	− 0.210	− 0.039
	*lnFD*	− 0.045	0.068	− 0.228	0.046	− 0.130	0.092	− 0.306	0.057
Test	R^2^	0.925				0.918			
	Adj-R^2^	0.907				0.899			
	AICc	229.907				246.470			
	Sum of squares of residuals	22.759				25.057			

**Figure 6:**
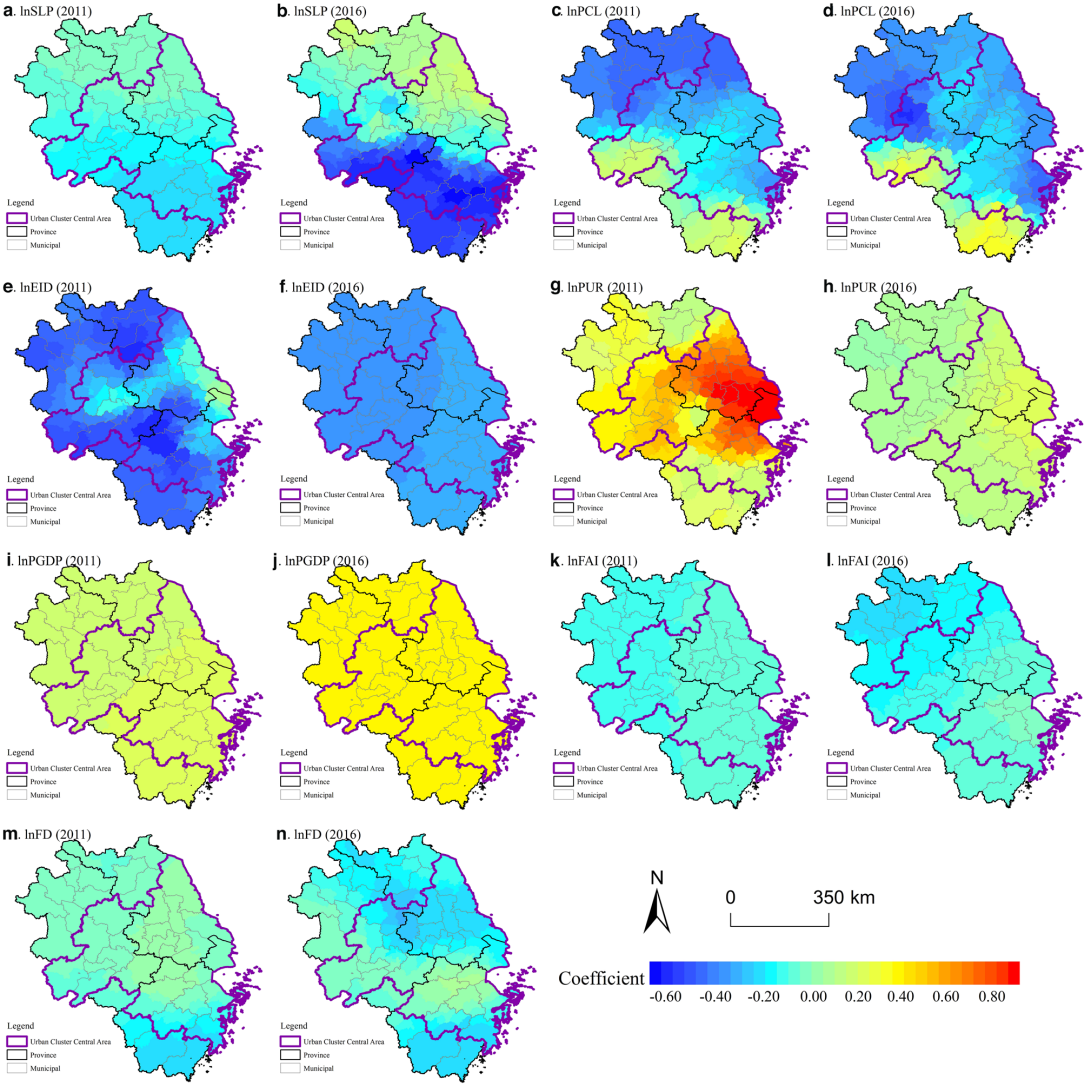
Spatial patterns of coefficients in the MGWR. (**a**) lnSLP(2011); (**b**) lnSLP(2016); (**c**) lnPCL(2011); (**d**) lnPCL(2016); (**e**) lnEID(2011); (**f**) lnEID(2016); (**g**) lnPUR(2011); (**h**) lnPUR(2016); (**i**) lnPGDP(2011); (**j**) lnPGDP(2016); (**k**) lnFAI(2011); (**l**) lnFAI(2016); (**m**) lnFD(2011); (**n**) lnFD(2016). Software: Arcgis 10.7. URL: https://www.esri.com/zh-cn/home.

The natural factors, including SLP, PCL, and EID, generally reflect the natural condition of the land that hosts human activities. It can be found from Table 5 that all-natural factors negatively affected LDI, which reflects the constraints on land development imposed by topographical conditions, and the importance of protecting arable land and the ecological environment under the national territorial spatial control. By further decomposing the effects of different factors in different geographical locations, we can see that the impact of SLP on LDI in 2011 and 2016 showed significant geographical divergence in space, with the areas of greater inhibition being distributed in Zhejiang Province and the southern Anhui, and the intensity of the effects gradually decreasing from the hilly areas of Zhejiang and Anhui to the plains of Jiangsu (Fig. 6a,b). It reflects that under the ecological civilization, land development is being carried out in a restrained manner based on nature conservation, and the development and construction activities in the YRD region should uphold the principle of ecological sustainability and rational use of land based on topographic conditions. The inhibiting effect of the PCL on LDI is stronger in the MAPZ such as northern Jiangsu and northern Anhui. The PCL is proportional to the LDI in the peripheral areas of the urban agglomeration of southern Anhui and Zhejiang, which are in the KEFZ. This reflects the fact that to a certain extent the pressure on arable land resources increases and construction activities are restricted (Fig. 6c,d). Thus, while providing land resources for construction activities, the focus should also be on protecting cropland through appropriate development and limiting excessive land construction. The areas where EID inhibited LDI with greater intensity are the KEFZs and MAPZs in northern Jiangsu and northern Anhui (Fig. 6e,f), and the global inhibitory effect gradually appeared by 2016. Due to the spatial control of the national territory, the awareness of the protection of ecologically important areas during land development has increased.

Human factors include PUR, PGDP, FAI, and FD. Among them, the PUR and PGDP had significantly positive impacts on LDI in YRD counties, while FAI and FD had a negative inhibitory effect which was deepening over time. As shown in Fig. 6g,h, the regression coefficient of the PUR was significantly positive but decreased from the central areas of urban agglomerations, especially the Shanghai and Nanjing metropolitan areas, the Taihu, and Hangzhou-Jiaxing areas, to the peripheral urban agglomerations. That is to say, the urban agglomeration is facing greater pressure for land development and construction due to a large population. In 2011, the positive effect of PGDP on LDI was more significant in coastal regions such as southern Jiangsu and Zhejiang than that in Anhui and northern Jiangsu (Fig. 6i); while in 2016, that across counties tended to be averaged (Fig. 6j), indicating that the global contribution of economic development to land development is further accentuated. At this stage, the government is no longer just carrying out construction activity under the concentration of economic investment, but more focusing on the development and utilization of its functional positioning, moving towards a development pattern of high quality and efficient utilization. The inhibitory effect of FAI on LDI decreased from the central areas of the eastern coastal urban agglomeration to the peripheral non-urban agglomeration regions (Fig. 6k). Combined with the industry structure of investment, it is easy to see that urban agglomerations always have obvious advantages in terms of investment attraction and policies, with fixed asset investments mainly in manufacturing and real estate industries flowing to these areas to obtain a higher rate of return on investment and construction. In contrast, non-central counties in northern Anhui, northern Jiangsu, and southern Zhejiang, based on the positioning of the MAPZ or KEFZ, have relatively concentrated public welfare investment, mainly in the water conservancy, environment and public facilities management industries and agriculture, which are usually not conducive to absorbing land development factors (Fig. 6l), thus leading to inhibiting effects and spatial variability. The effect of FD on LDI depended on the actual situation of the counties. In Hangzhou, Nanjing, and Su-Xi-Chang metropolitan areas, the impact of FD on LDI was positive (Fig. 6m), while in contrast, the suppression effect was significant in the highly UZ of the Nanjing metropolitan area and the KEFZ of southern Zhejiang (Fig. 6n). It shows that local governments can attract more environmental industries through financial regulation and policy control, raise the threshold of access to land construction industries, and reasonably restrict excessive development to achieve the intensive use of national land space.

#### Differences in driving factors between functional zones

**Figure 7:**
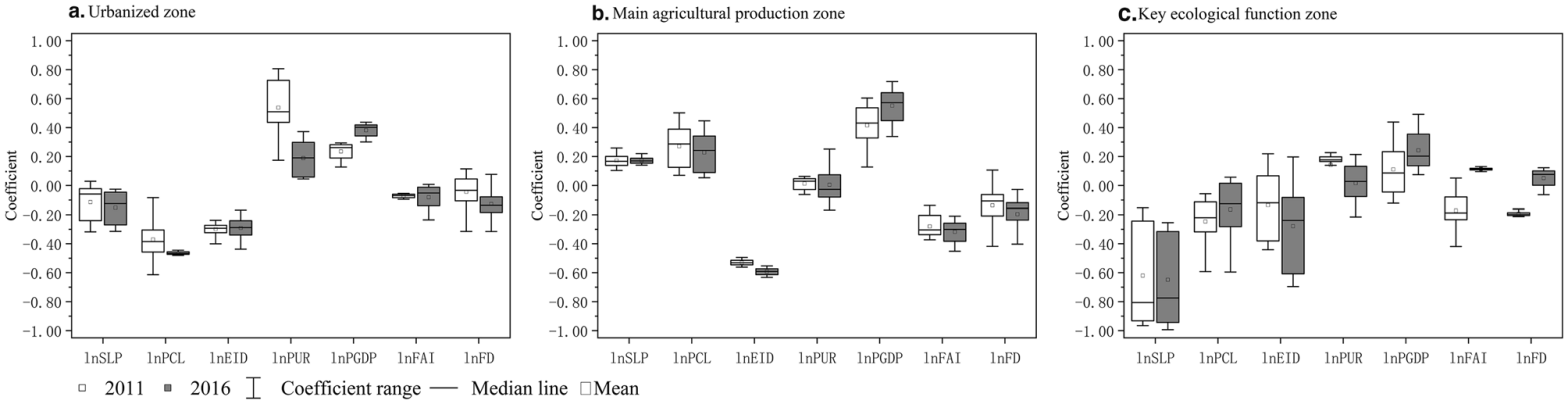
Regression coefficients in MGWR of major functional zones. (**a**) Urbanized zone. (**b**) Main agricultural production zone. (**c**) Key ecological function zone. Software: Origin 2018. URL: https://www.originlab.com/.

As shown in Table 6 and Fig. 7, the drivers of FDI within different functional zones, i.e., UZ, MAPZ, and KEFZ, have been estimated separately. The UZ includes Shanghai, Nanjing, Hangzhou, and Ningbo, the Su-Xi-Chang metropolitan areas, the counties along the rivers, and the main urban counties of each city. The overall average LDI of the region was over 33%, much higher than the others, making it a key area for land development across the YRD. The region has a flat topography, a high level of economic development, a high degree of openness based on the advantages of the coast, a gradually improving town system, and a certain degree of radiation drive from the cities. The regression coefficients of the drivers of each sub-model show that the LDI in UZ was determined by both natural-human factors, with the PUR and PGDP contributing more. It is also closely related to the larger economic scale and more robust town system in UZ. The inhibiting effect of natural factors on land development fully reflects those natural endowments are the basis for human activities. The PCL coefficient has the highest absolute value and the strongest negative effect, indicating the scarcity of arable land resources in UZ. By 2016, the negative impact of FAI and FD in the sample of UZ had deepened significantly, reflecting that the region is at a more mature stage of urban integration development. Promote a shift in industrial structure towards high value-added, high-end industries through changes in fixed asset input industries and increased fiscal freedom. There has been a fundamental shift in optimizing economic development to reduce energy consumption and environmental pollution.

**Table 6:** The statistical description of MGWR sub-model.

Variables	the YRD		UZ		MAPZ		KEFZ	
	2011	2016	2011	2016	2011	2016	2011	2016
lnSLP	− 0.241***	− 0.263***	− 0.252***	− 0.308***	0.174	0.062	− 0.526***	− 0.573***
lnPCL	− 0.361***	− 0.341***	− 0.465***	− 0.516***	0.372***	0.291**	− 0.233***	− 0.018
lnEID	− 0.456***	− 0.439***	− 0.230***	− 0.243***	− 0.543***	− 0.599***	− 0.336***	− 0.250**
lnPUR	0.277***	0.111***	0.367***	0.131***	0.148	0.003	0.152**	− 0.074
lnPGDP	0.120***	0.237***	0.095*	0.217***	0.300	0.415**	0.083	0.346***
lnFAI	− 0.128***	− 0.182***	− 0.074*	− 0.156***	− 0.290***	− 0.373***	− 0.166**	0.072
lnFD	− 0.169*	− 0.244***	− 0.082*	− 0.170***	− 0.105	− 0.263*	− 0.141	0.084
*Adj R*^*2*^	*0.907*	*0.899*	*0.826*	*0.790*	*0.728*	*0.749*	*0.904*	*0.894*
*AICc*	*229.907*	*246.470*	*249.034*	*273.428*	*152.557*	*148.916*	*79.432*	*84.928*

The MAPZ is dominated by non-main urban counties such as northern Jiangsu and northern Anhui, where the LDI was 15.60% in 2011 and 16.22% in 2016. The terrain is suitable for cultivation and has good conditions for agricultural production. The LDI in the region is mainly driven positively by the PCL, with prominent disincentives from EID, FAI, and FD. The objectives of the region are to restrict large-scale, high-intensity industrialization and urbanization in the development of land space, and the important ecological farming requirements limit the occupation of land by construction activities. The region is subject to less pressure on arable land resources, and fixed asset investment is tilted from real estate and new district construction inputs to farming industries such as agriculture, forestry, animal husbandry, and fishing. By 2016, finance is gradually playing a role in the development of land in the MAPZ, and the guidance of agricultural development inputs in the fiscal plan based on functional positioning may limit the construction of regional urbanization to ensure the development capacity of agricultural products.

The KEFZ includes the low hill counties of southern Anhui and western Zhejiang. The area has a high average slope, with some natural beauty of the landscape, and the lowest overall LDI, at 5.56% and 6.01% in 2011 and 2016 respectively. The influence of the drivers reflects the fact that the region is mainly inhabited by SLP, and EID and driven by PGDP. Due to the topography and the requirements of ecological construction, the LDI in the area should be strictly limited, while the PGDP rises to a certain level, the requirements of the government or residents for the quality of the ecological environment prompt strong investment in green industries and promote the development and upgrading of environmental protection technology projects to protect and restore the ecological environment.

## Discussion

Unlike single-scale studies^11,12,19^, we explore the LDI from a multi-scale of “provincial-municipal-county-functional zone”. We find that different levels present different land development characteristics. The results in the provincial and municipal scales allow for a better summary of regional differences and the precise formulation of development objectives based on the natural foundation and positioning of the region^8,34^; while the LDI in micro-county and functional zone levels reflect the implementation of heterogeneous and functional zones in urban agglomerations. The county level, due to its small scope, provides a more intuitive insight into the microscopic phenomena that exist in land development^15^, such as the development pattern along the river axis and the core–edge mode. The major functional zone level can reflect the differences within each area and is important for observing the effectiveness since the implementation of MFZP.

Deconstructing the drivers of LDI at the global and sub-functional scales separately can reflect the regional and intra-regional drivers interactively. Whereas studies at a global scale can only show macroscopic influences, zonal models are more accurate in identifying influences in different regions^13,14^. We found that from the global scale, most of the natural and human factors will have an impact on LDI, and they are not representative^10^, but the factors at the major functional zone level are more accurate. We further discussed the reasons behind each driver factor and found that firstly under the establishment of an ecological civilization, the land is being developed in a constrained manner to protect nature and is being used rationally because of its original topography and ecological importance. Secondly, the large urban population and economic activities will increase the demand for land, and the regulation of population size according to the carrying capacity and development potential promotes the healthy development of the national land development. In addition, economic development promotes the expansion of space for construction activities, especially along the coast and along the rivers and metropolitan areas. Most importantly, when the LDI increases, the constraints on available land resources tighten, and FAI shifts towards positioning functional industries. The increased level of financial input has given local governments greater financial autonomy, awareness of land resource protection has increased, and the government has exercised fiscal regulation to limit the over-development of land.

Binding mechanism and functional differences, we should still insist on and adopt the implementation of zoning control. Adapting to local conditions is an important principle for sustainable land development^31,32,35^. Firstly, the UZ is at a more mature stage of urban integration development. It should take advantage of its location to drive the land construction in the surrounding counties. The financial advantages also should be used to enhance the gathering capacity of industries. A fundamental transformation of the economic development mode should be optimized to the goal of reducing energy consumption and ecological pollution. Secondly, the MAPZ aims to restrict large-scale and high-intensity industrialized and urbanized development in the spatial development of the country. Thus, the guidance of agricultural development inputs in the fiscal plan restricts the construction to ensure the development capacity of agricultural products in the region. Thirdly, subject to the requirements of topography and ecological construction, the KEFZ should strictly limit the LDI and invest heavily in forestry as well as green industry investment in environmental technology projects to protect the environment.

## Conclusions

We jointly describe the spatial differentiation characteristics of LDI in more detail at multiple scales, and such interaction can provide a basis for cross-administrative cooperation and urban planning nesting at different administrative levels. Specifically, the development of provincial and municipal level land construction is mainly influenced by natural conditions, showing a gradient of development from Shanghai > Jiangsu > Anhui > Zhejiang in decreasing order. The urban agglomeration in the YRD plays a leading role in the development of the entire territory. The LDI at the county level is on the rise and varies significantly in time and space, showing a point-axis development pattern with Shanghai as the core along the river as the axis of the YRD, and a core–edge development pattern within each city.

The premise of scientific urban planning is to excavate the driving force and braking force of urban development. The estimation results at the global scale prove that LDI can be affected by many natural and human factors. Since the implementation of the MFZP, the functional zones have been positioned and the uses of land development match the development aims. It can be found that the LDI decreases in a gradient of UZ > MAPZ > KEFZ. Densely populated UZ with rapid economic development is influenced by conforming factors such as SLP, PCL, EID, PUR, PGDP, FAI, and FD. In the MAPZ, the LDI is mainly influenced by the facilitating role of PCL, EID, FAI, and the prominent role of FD. In the KEFZ, the LDI is mainly influenced by the inhibiting effect of natural factors SLP, and EID and driven by PGDP.

Hence, we propose the following policy implications to facilitate the development and construction of regions in a more differentiated and precise manner. (1) Due to the characteristics of regional differences, the multi-scale articulation of the constraint policy of LDI from the central to the local level should be strengthened. For instance, the strong areas in UR may get more construction land development indicators beyond their carrying capacity, while weak areas are deprived of development opportunities. (2) Focusing on the integration of objectives and process control, that is, determining the upper limit of development according to the LDI, and then determining the lower limit of the minimum protection space to prevent development behavior from breaking through the reverse constraint. (3) Using a combination of policy tools and a system of rewards and punishments, that is, incentivizing inhibitory factors (e.g., EDI) to improve the efficiency of ecological protection and punishing positive factors for over-exploitation of land. (4) Further refining the direction of regulation and control of the three functional zones. On the one hand, UR should determine the development intensity reasonably in conjunction with the land carrying capacity, and implement strict total control indicators. On the other hand, the region should focus on the transformation from land development scale to land development efficiency, and strengthen the consideration of land use efficiency assessment indicators. MAPZ and KEFZ should strengthen the spatial landing of rigid constraints on land development behavior by delineating permanent basic farmland and ecological protection red line. In addition, ecological compensation should be strengthened for the deprivation of development rights and interests caused by arable land protection and ecological protection.

In the future, we can further strengthen the multi-scale analysis and add more factors and conduct longer time series follow-up studies to observe the specific impact of different stages of the implementation of the MFZP, perhaps using panel data (e.g., 2010–2020), or using more five-year period data (e.g., 2010, 2015, 2020).

## Data Availability

In the current study, socio-economic data is available through China's statistical yearbooks at all levels, and land data is not publicly available due to administrative regulations.
